# Pandemic distress associated with segregation and social stressors

**DOI:** 10.3389/fpubh.2023.1092269

**Published:** 2023-03-24

**Authors:** Rodman Turpin, Salvatore Giorgi, Brenda Curtis

**Affiliations:** ^1^Department of Global and Community Health, College of Public Health, George Mason University, Fairfax, VA, United States; ^2^National Institute on Drug Abuse, National Institutes of Health, Bethesda, MD, United States

**Keywords:** race, segregation, social support, stress, bias

## Abstract

**Background:**

Racial/ethnic minorities are disproportionately impacted by the COVID-19 pandemic, as they are more likely to experience structural and interpersonal racial discrimination, and thus social marginalization. Based on this, we tested for associations between pandemic distress outcomes and four exposures: racial segregation, coronavirus-related racial bias, social status, and social support.

**Methods:**

Data were collected as part of a larger longitudinal national study on mental health during the pandemic (*n* = 1,309). We tested if county-level segregation and individual-level social status, social support, and coronavirus racial bias were associated with pandemic distress using cumulative ordinal regression models, both unadjusted and adjusted for covariates (gender, age, education, and income).

**Results:**

Both the segregation index (PR = 1.19; 95% CI 1.03, 1.36) and the coronavirus racial bias scale (PR = 1.17; 95% CI 1.06, 1.29) were significantly associated with pandemic distress. Estimates were similar, after adjusting for covariates, for both segregation (aPR = 1.15; 95% CI 1.01, 1.31) and coronavirus racial bias (PR = 1.12; 95% CI 1.02, 1.24). Higher social status (aPR = 0.74; 95% CI 0.64, 0.86) and social support (aPR = 0.81; 95% CI 0.73, 0.90) were associated with lower pandemic distress after adjustment.

**Conclusion:**

Segregation and coronavirus racial bias are relevant pandemic stressors, and thus have implications for minority health. Future research exploring potential mechanisms of this relationship, including specific forms of racial discrimination related to pandemic distress and implications for social justice efforts, are recommended.

## Introduction

Racial/ethnic minorities have faced significant health disparities related to the COVID-19 pandemic, including a greater burden of COVID-19 related hospitalization and death, depression, anxiety, financial strain, and housing insecurity (1–13). Additionally, racial/ethnic minorities have experienced significant racial discrimination related to COVID-19, including harassment, employment discrimination, and violence (14–17). COVID-19 related experiences of racism are particularly pronounced towards Asian-Americans, with one study finding that one in five Asian Americans had direct experiences with overt discrimination, such as physical attacks and xenophobic slurs (18, 19). The experience of these race-related stressors can have important implications for many adverse health outcomes among racial/ethnic minorities.

Minority stress theory, while originally and most commonly focused on sexual minorities (20), has expanded in use to apply to several minoritized groups, including racial/ethnic minorities (21). Racial/ethnic minorities also experience additional social and structural stressors compared to their non-minority counterparts, which can lead to adverse health outcomes. Among racial/ethnic minority groups, this can include interpersonal forms of racism such as being verbally threatened, harassment, and slurs, as well as structural forms of racism such as institutional discrimination, over-policing, and segregation. Interpersonal racism in particular has exacerbated in the context of the Covid-19 pandemic, as growing literature demonstrates that experiences of coronavirus-related racial bias are particularly prevalent among Asian and Black individuals (19, 22–28). Much like racial discrimination and bias in other contexts, these experiences can lead to several adverse mental health outcomes, including depression, anxiety, suicidality, and substance use, which is closely linked to mental health.

Regarding structural factors, racial segregation is an especially impactful form of structural racism associated with numerous adverse mental and physical health outcomes (29–33). Minority communities, particularly Black communities, are disproportionately marginalized through both historical and current segregation (29–32). Segregation is associated with substantially more vulnerability to Covid-19 through several mechanisms, including increased household density, reliance on crowded transportation services, and lower access to healthcare services (34–38). Additionally, segregation exacerbates socioeconomic difficulties through limited access to employment and educational opportunities (34–36). For households in segregated neighborhoods, it is often much more difficult to recover from sudden socioeconomic challenges related to the pandemic, such as healthcare costs or the loss of employment. In tandem, coronavirus-related racial bias and segregation can lead to substantial pandemic distress, and thus have implications for mental and physical health disparities across race/ethnicity.

Social support can be an important buffer against the adverse effects of minority stressors, as there is a wealth of literature demonstrating the benefits of social support in protecting against many adverse mental health outcomes (20, 39–41). Social capital theory posits that social relationships are important resources that can allow for accumulation of capital, including a greater means of coping with adversity (42). When facing difficulties related to the COVID-19 pandemic, social support can provide a greater sense of social cohesion, as well as tangible support for facing socioeconomic challenges related to the pandemic. Additionally, having a stronger sense of social status in one’s community can reflect greater social capital, allowing for better community connectedness and more effective coping with challenges. Both social support and social status can be important protective factors against COVID-19 related adversity (20, 39–43).

**Figure 1:**
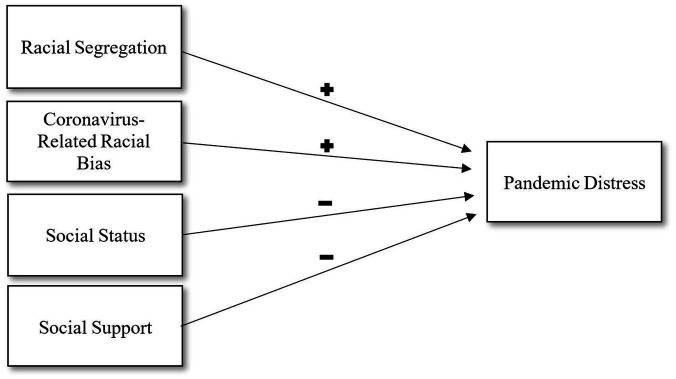
Directed acyclic graph for relationships between racial segregation, coronavirus-related racial bias, social status, social support, and pandemic distress. Positive signs indicate hypohesized negative relationships.

Based on this, the purpose of our study was to test for associations between pandemic distress outcomes and four exposures: racial segregation, coronavirus-related racial bias, social status, and social support. We hypothesized that racial segregation and coronavirus-related racial bias would be associated with greater pandemic distress, and that higher social status and social support would be associated with lower pandemic distress (Figure 1).

## Methods

### Sample

Data for the current study consists of a sample of participants recruited as part of a national, longitudinal study on COVID-19, substance use, and mental health (44). Consenting participants were recruited online *via* a Qualtrics Panel and met the following requirements: (1) lived in the U.S., (2) were at least 18 years old, (3) wrote at least 500 words across their Facebook status timeline, and (4) posted at least 5 posts within the 180 days prior to recruitment into our study. While Facebook use was a requirement for the larger study, no Facebook data is used in the current study. Data was collected between September 2020 and June 2021. The current sample consists of *n* = 1,309 participants. This study was considered exempt by the University of Pennsylvania’s institutional review board.

### Key variables

Our key exposures of interest included segregation, coronavirus-related racial bias, social status, and social support. County-level segregation was measured using the residential segregation index from the U.S. Census American Community Survey 5-year estimates (2016–2020), obtained from the 2020 County Health Rankings. Residential segregation is a dissimilarity index, which measures the percentage of the population within each census tract (i.e., sub-county spatial unit) which would need to change residence to have the same racial demographic percentage as the county overall. Coronavirus-related racial bias was measured using the 9-item Coronavirus Racial Bias Scale (CRBS); and is used to assess beliefs about how COVID-19 negatively affecting attitudes toward one’s race/ethnicity (Cronbach’s alpha = 0.87) (25). Each item is measured on a 4-point Likert scale where options range from 1 (Strongly disagree) to 4 (Strongly agree). Social status was measured using the single item MacArthur Scale of Subjective Social Status, which asks participants to evaluate where they stand within society (29). This single item uses a 10-point scale, visualized as a ladder, where 1 represents those who are the worst off in society (i.e., the least education and income) and 10 represents those best off in society (i.e., the most education and income). Social support was measured using the 6-item Perceived Social Support scale (F-SozU K-6) (45). All items (e.g.,” I receive a lot of understanding and security from others”) are measured on a 5-point Likert scale (1 = Strongly disagree and 5 = Strongly agree) (Cronbach’s alpha = 0.86).

Our key outcome was pandemic stress, measured using the Pandemic Stress Index (PSI) (46). For bivariate analyses, this index was dichotomized for ease of presentation and interpretability. For regression analyses, we used this index in its original continuous form (Cronbach’s alpha = 0.89).

### Other variables

Other variables included gender (man, non-binary, woman), age (18–29, 30–39, 40–49, 50 or older), race (Asian/Pacific Islander, Black, White, Other), Ethnicity (Hispanic/Latino, Non-Hispanic Latino), highest education level (High School or less, Some College or Trade School, Two-Year College Degree, Four Year College Degree or more) and annual household income (Less than $30,000, $30,000 to $59,999, $60,000 to $89,999, $90,000 or more).

### Missing data

Missingness was overall low for all items (<5%). We conducted intrascale stochastic imputation to impute missing observations for the coronavirus racial bias scale, the social, support scale, and the social status scale. This was appropriate given the low nonresponse for all variables, and good internal consistency of the items (Cronbach’s alpha >0.80 for all items).

### Bivariate analyses

For bivariate analyses, we tested for differences in a dichotomized pandemic distress measure (dichotomized at its median) across all of our key measures (Racial segregation, coronavirus racial bias, social status, and social support) using Cochran-Armitage tests of trend. Ordinal tests were used due to the non-normality of each of these continuous measures. We also tested associations between pandemic distress and our covariates using a Chi-Square test for binary/multicategorical covariates (gender, race, ethnicity), and a Cochran-Armitage test for ordinal covariates (age, education, income).

### Regression analyses

We tested for associations between each of our four key factors and the continuous pandemic distress outcomes using ordinal regression models. Unadjusted cumulative ordinal prevalence ratios were generated testing associations between each factor and pandemic distress. We also generated adjusted ratios using a single model containing all four measures and terms for gender, age, education level, and annual household income. Note that we do not include race or ethnicity as covariates since our main exposures of interest include racism; not only is it conceptually flawed to examine racism independent of race, but analytically the collinearity between race/ethnicity and racism prevents the use of both measures in the same regression model.

### Quality assurance and statistical software

We tested regression models for collinearity by measuring the variance inflation factor (VIF) in all models: There was no evidence of collinearity (all VIF < 5) for any of the terms included in the final models. We identified no influential outliers using Leverages and Cook’s distances. All analyses were conducted using SAS 9.4 (47).

## Results

### Sample characteristics

Our analytic sample consisted of 72.4% women, 25.8% men, and 1.8% non-binary participants (Table 1). The sample was 11.7% Black, 8.6% Asian, and 11.6% Hispanic. Examining socioeconomic status, 26.1% of the sample had a 4-year degree or higher, and 52.2% had a household income of $60,000 or more. The median scores for our key variables (all scaled in percentage, ranging form 0% minimum to 100% maximum) were 59% for segregation, 14% for coronavirus racial bias, 55% for social status, and 75% for social support.

**Table 1:** Sample characteristics and bivariate associations with pandemic distress among an internet-based sample (*n* = 1,309).

	Total		Low pandemic distress	High pandemic distress	*p* value
**Categorical/ordinal measures**					
Gender^1^	*n*	%	%	%	0.0117
Man	338	25.8	26.6	23.4	
Non-Binary	24	1.8	1.2	3.7	
Woman	947	72.4	72.1	73.0	
Age group^2^					0.1658
18–29	350	26.7	25.8	29.5	
30–39	413	31.6	31.8	30.7	
40–49	273	20.9	20.6	21.6	
50 or older	273	20.9	21.7	18.2	
Race^1^					<0.0001
Asian/pacific islander	112	8.6	8.7	8.2	
Black	153	11.7	10.7	14.6	
Other	63	4.8	3.4	9.1	
White	981	74.9	77.2	68.1	
Ethnicity^1^					0.0004
Hispanic/Latino	152	11.6	9.8	17.0	
Non-Hispanic/Latino	1,157	88.4	90.2	83.0	
Highest education level^2^					0.0872
High school or less	100	7.6	8.5	5.2	
Some college or trade school	354	27.0	26.7	28.0	
Two-year college degree	513	39.2	40.0	36.8	
Four year college degree or more	342	26.1	24.8	30.1	
Annual household income^2^					0.0019
Less than $30,000	272	20.8	18.0	29.2	
$30,000 to $59,999	354	27.0	28.4	23.1	
$60,000 to $89,999	281	21.5	21.6	21.0	
$90,000 or more	402	30.7	32.0	26.8	
**Indices/scales**	*n*	**Median** ^**3**^	**Median** ^**3**^	**Median** ^**3**^	
Social status index %^2^	1,309	55.0 (0.22)	55.5 (0.22)	44.4 (0.33)	<0.0001
Social support index %^2^	1,309	75.0 (0.33)	79.1 (0.29)	70.8 (0.38)	<0.0001
Segregation index %^2^	1,309	59.0 (0.23)	48.7 (0.21)	62.8 (0.24)	0.0115
Coronavirus racial bias scale %^2^	1,309	14.8 (0.37)	11.1 (0.33)	18.5 (0.40)	0.0401

1 Tested using Chi-square test.

2 Tested using Kruskal–Wallis test.

3 Interquartile range provided in parentheses.

### Bivariate results

We observed differences in all of our key measures across dichotomized levels of pandemic distress. Participants with higher pandemic distress were characterized by higher median scores for segregation (62.8% compared to 48.7%) and coronavirus racial bias (18.5% compared to 11.1%), as well as lower scores for social status (44.4% compared to 55.5%) and social support (70.8% compared to 79.1%). Higher pandemic distress was also associated with lower income, non-binary gender, and Black, Hispanic, and Other racial identities.

### Regression results

All of our key variables were significantly associated with pandemic distress, both before and after adjustment for covariates (Table 2). Higher prevalence of pandemic distress was associated with greater segregation (aPR = 1.15, 95% CI 1.00, 1.31), greater coronavirus racial bias (aPR = 1.12, 95% CI 1.02, 1.24), lower social status (aPR = 0.74, 95% CI 0.64, 0.86), and lower social support (aPR = 0.81, 95% CI 0.73, 0.90). Additionally, non-binary gender (aPR = 1.22, 95% CI 1.02, 1.46) and higher education (aPR = 1.23, 95% CI 1.11, 1.36) were both associated with greater pandemic distress.

**Table 2:** Segregation, coronavirus racial bias, and covariates associated with pandemic distress among an internet-based sample (*n* = 1,309).

	Unadjusted			Adjusted (for all terms with included estimates)		
	PR	Lower CI	Upper CI	PR	Lower CI	Upper CI
Segregation index %	**1.19**	**1.03**	**1.36**	**1.15**	**1.00**	**1.31**
Coronavirus racial bias scale %	**1.17**	**1.06**	**1.29**	**1.12**	**1.02**	**1.24**
Social status index %	**0.64**	**0.50**	**0.81**	**0.74**	**0.64**	**0.86**
Social support index %	**0.79**	**0.63**	**0.98**	**0.81**	**0.73**	**0.90**
**Gender**						
Man				Ref.	-	-
Non-binary				**1.22**	**1.02**	**1.46**
Woman				1.03	0.98	1.09
**Age group**						
18–29				Ref.	-	-
30–39				0.97	0.91	1.03
40–49				0.99	0.93	1.06
50 or older				0.97	0.91	1.04
**Highest education level**						
High school or less				Ref.	-	-
Some college or trade school				**1.12**	**1.02**	**1.23**
Two-year college degree				**1.15**	**1.05**	**1.26**
Four year college degree or more				**1.23**	**1.11**	**1.36**
**Annual household income**						
Less than $30,000				Ref.	-	-
$30,000 to $59,999				0.91	0.83	1.00
$50,000 to $89,999				0.95	0.88	1.03
$90,000 or more				0.95	0.87	1.02

Bolded estimates and intervals have a value of *p* < 0.05. PR, prevalence ratio; CI, confidence interval; Ref, reference group.

## Discussion

We found that racial segregation and coronavirus-related racial bias were associated with greater pandemic distress, and that social status and social support were associated with lower pandemic distress. This is consistent with much of the overall literature demonstrating that racism, in several structural and interpersonal forms, creates a greater stress burden on racial minorities (14–17, 19, 22, 23, 25–28, 30, 31). In the context of the COVID-19 pandemic, segregation can create a greater stress burden in several ways, but most notably socioeconomic marginalization from access to quality employment, increased housing density due to crowding, and limited healthcare access. Each of these factors can lead to incredible economic and mental health strain. Interpersonal experiences of coronavirus-related racial bias can also lead to poorer social cohesion, a lack of access to community services, and greater vulnerability to race-related violence. Overall, these both represent impactful forms of racism that directly produce health disparities burdening racial/ethnic minorities.

Racial segregation reinforces health disparities by limiting access to resources that impact health—such as education, income, and occupation (32–38). These limitations were exacerbated during Covid-19 where individuals of color who have experienced a lifetime of exposure to racial segregation also experienced worse quality of and access to health care and social support (32, 36). Additionally, racial segregation may create an environment that heightens stress related to discrimination, both experienced and anticipated (e.g., coronavirus racial bias) which can worsen pandemic distress. This can have significant implications for the mental and physical health of racial/ethnic minorities, as these stressors may not only exacerbate depression, anxiety, and other adverse mental health outcomes, but may also affect physical health, such as cardiovascular disease which is well-documented as a long-term outcome of stress (48). Pandemic stress may worsen many health disparities among racial/ethnic minorities compared to their non-minority peers.

Our findings highlight the importance of social status and social support in potentially mitigating pandemic distress. Social support could protect against pandemic strain in several different ways, such as having a network for family and friends that can assist with household needs while one is recovering from COVID-19, feeling less isolated, and having more socioeconomic support to manage the direct and indirect costs of healthcare. A sense of social status in one’s community often reflects a level of community connectedness that may confer these benefits as well. In these ways, social status and support can be directly beneficial to the health of racial/ethnic minorities navigating the challenges of the pandemic (20, 49).

Strengths of our study include a large dataset covering a wide range of participants across race/ethnicity, gender, and age. We utilized both a more general measure of structural racism (segregation) as well as a measure of racism specific to the pandemic (coronavirus-related racial bias), making our findings especially contextually appropriate. Our measures also capture several items covering different dimensions of each key construct, making our overall findings more comprehensive. Finally, our work fills an important gap on a relatively understudied, yet particularly salient topic. Given that the pandemic is still ongoing, and racial inequities in several aspects of health persist, our findings add to a significant area of the literature.

Our research has limitations that should be acknowledged. The use of self-reported measures of social status and social support largely reflect self-perceptions, and thus may differ substantially from social network and community contexts. Despite this limitation, the perceptions of one’s social support and status are still quite relevant to health and may be even more predictive of one’s mental and emotional health than measures of network and community structure. While we identified significant associations between non-binary gender and greater pandemic stress, this represented a very small proportion of our sample, so it is difficult to generalize. Future research exploring pandemic stress in larger studies of non-binary people is recommended. The restriction to Facebook users does limit the generalizability of the study, as internet users broadly are generally younger and more socioeconomically advantaged than the general population, as more socioeconomically disadvantaged and older populations may not be fully captured. Our study sample demographics are fairly similar to the U.S. population across age and education however, though it is more predominantly female than the general population (50). Finally, social desirability bias is likely to impact the measures used in our study, particularly in the self-reported measures of social status, social support, and coronavirus-related racial bias. Notably however, this does not impact our measure of segregation, as it was not self-reported.

## Conclusion

Racial segregation and coronavirus-related racial bias were both associated with greater pandemic distress, while social status and social support were associated with lower pandemic distress. Future research exploring mechanisms of these relationships, such as specific outcomes of segregation that create a greater stress burden on racial minorities, is recommended.

## Data availability statement

The anonymized data analyzed in this study is subject to the Creative Commons Attribution-NonCommercial-NoDerivatives 4.0 International (CC BY-NC-ND) and made publicly available via OSF at https://osf.io/sa76w/.

## Author contributions

RT: conceptualization, methodology, writing- original draft preparation. SG: data curation, writing-reviewing and editing. BC: conceptualization, supervision, writing-reviewing and editing. All authors contributed to the article and approved the submitted version.

## Funding

This research was supported by the Intramural Research Program of the NIH, NIDA. Rodman Turpin is also supported by the National Institute on Minority Health and Health Disparities (K01MD016346) and the University of Maryland Prevention Research Center cooperative agreement from the Centers for Disease Control and Prevention (U48DP006382). Any interpretations and opinions expressed herein are solely those of the authors and may not reflect those of NIMHD or the CDC.

## Conflict of interest

The authors declare that the research was conducted in the absence of any commercial or financial relationships that could be construed as a potential conflict of interest.

## Publisher’s note

All claims expressed in this article are solely those of the authors and do not necessarily represent those of their affiliated organizations, or those of the publisher, the editors and the reviewers. Any product that may be evaluated in this article, or claim that may be made by its manufacturer, is not guaranteed or endorsed by the publisher.
